# Nutrient Sensing Kinases PKA and Sch9 Phosphorylate the Catalytic Domain of the Ubiquitin-Conjugating Enzyme Cdc34

**DOI:** 10.1371/journal.pone.0027099

**Published:** 2011-11-07

**Authors:** Ross Cocklin, Mark Goebl

**Affiliations:** Department of Biochemistry and Molecular Biology, Indiana University School of Medicine, Indianapolis, Indiana, United States of America; University of Minnesota, United States of America

## Abstract

Cell division is controlled in part by the timely activation of the CDK, Cdc28, through its association with G1 and G2 cyclins. Cdc28 complexes are regulated in turn by the ubiquitin conjugating enzyme Cdc34 and SCF ubiquitin ligase complexes of the ubiquitin-proteasome system (UPS) to control the initiation of DNA replication. Here we demonstrate that the nutrient sensing kinases PKA and Sch9 phosphorylate S97 of Cdc34. S97 is conserved across species and restricted to the catalytic domain of Cdc34/Ubc7-like E2s. Cdc34-S97 phosphorylation is cell cycle regulated, elevated during active cell growth and division and decreased during cell cycle arrest. Cell growth and cell division are orchestrated to maintain cell size homeostasis over a wide range of nutrient conditions. Cells monitor changes in their environment through nutrient sensing protein kinases. Thus Cdc34 phosphorylation by PKA and Sch9 provides a direct tether between G1 cell division events and cell growth.

## Introduction

The ubiquitin proteasome system (UPS) controls cellular functions through the targeted degradation of key regulatory proteins. The covalent attachment of ubiquitin often serves as a signal for the degradation of these regulatory proteins by the 26S proteasome (for review see [1]). The first step in ubiquitylation is the formation of a high energy intermediate between ubiquitin and a conserved cysteine of the ubiquitin activating (or E1) enzyme. E1 then transfers the ubiquitin via a thiolester linkage to a conserved cysteine of an ubiquitin conjugating (or E2) enzyme. The final transfer of ubiquitin to a specific substrate typically requires both an activated E2 as well as a particular ubiquitin ligase (E3), which provides specific substrate modifying capacity, forming an isopeptide linkage between the COOH-terminal glycine residue of ubiquitin and the ε-amino group of a lysine residue of the substrate. A substrate is often targeted for degradation upon the addition of a polyubiquitin chain to the lysine 48 residue of ubiquitin.

*CDC34* encodes a ubiquitin conjugating enzyme that is essential for cell viability and the initiation of DNA replication in the yeast, *Saccharomyces cerevisiae* [2]. Cdc34 conjugates ubiquitin with target proteins in conjunction with the SCF family of E3-ubiquitin ligases [3]. A functional SCF complex consists of at least four distinct proteins, Skp1, Cdc53, Rbx1 and an F-box protein, the component that determines substrate specificity (for review, see [4]). When Cdc4 is present in the SCF complex, Cdc34 and SCF^Cdc4^ mediate ubiquitylation and subsequent degradation of the cyclin dependent kinase inhibitors Sic1 and Far1 [5], [6], [7]. On the other hand, Cdc34 and SCF^Grr1^ ubiquitylate the cyclins Cln1 and Cln2 [6], [7], [8].

The Cdc34/Ubc7 family of ubiquitin conjugating enzymes is defined by a conserved motif within the catalytic domain that consists of two serines and a twelve amino acid acidic loop, which lie in close physical proximity to the catalytic cysteine. In contrast, the majority of E2s, of which Rad6 is a classic example, have a lysine and aspartic acid residue in lieu of these serine residues and lack the acidic loop. This motif allows the Cdc34/Ubc7 family to catalyze both monoubiquitylation and ubiquitin chain extension [9]. Accumulating evidence suggests the formation of the Cdc34∼ubiquitin thiolester precedes self-association of Cdc34, which in turn is critical for Cdc34 catalytic activity [10], [11]. Interestingly, Cdc34^S97D^ mutants are unable to homodimerize and are nearly inviable [11]. Elegant *in vitro* reconstitution of Sic1 polyubiquitylation by SCF^Cdc4^ has demonstrated that conjugation of the first ubiquitin to the substrate is the rate limiting step in this process. *In vitro*, Cdc34 mutants that lack the acidic loop (Cdc34^Δ12^) monoubiquitylate Sic1 with kinetics comparable to wild type Cdc34, but extend ubiquitin chains at a negligible rate [9]. However, during *in vitro* Cdc34 autoubiquitylation or histone ubiquitylation assays which do not require RING finger containing proteins, Cdc34^Δ12^ mutants function as well as, if not better than, Cdc34 [11], [12]. Cells solely expressing Cdc34^Δ12^ mutants are nearly inviable, as are cells harboring Cdc34^S97D^ or Cdc34^S73K/S97^ mutants [13]. Paradoxically, deletion of the acidic loop, residues 103–114, in combination with S73K and S97D mutations (hereafter referred to as the Cdc34 triple mutant, Cdc34^tm^) shows only subtle defects on cell growth *in vivo* [11], [12], [13]. However, *in vitro* SCF^Cdc4^ dependent Cdc34^tm^ polyubiquitylation of Sic1 is defective similar to Cdc34^Δ12^ [14]. Recent data indicates that Cdc34^tm^ expressing cells show key differences from wild-type cells. Importantly, Cln1 and Cln2 proteins are more stable, while Sic1 has a decreased t_1/2_ and Far1 becomes undetectable. Further, the steady state level of the Ace2 and Swi5 transcription factors, as well as the abundance of their transcriptional targets, is altered. A subsequent Synthetic Gene Array (SGA) screen revealed that *CDC34* ^tm^ cells depend on the expression of *RAD23*, *RPN10* and several other regulators of the UPS [15]. Interestingly, loss of the Cdc34/Ubc7 specific motif causes cells to become dependent on Cka2 and Ubp14, most likely due to an increase in toxic, free ubiquitin chains [14], [15].

This study demonstrates that the Cdc34/Ubc7 specific motif is also a key target of signaling pathways coordinating the regulation of cell growth in response to changes in environmental conditions such as nutrient levels. Here we demonstrate that Cdc34-S97 can be directly phosphorylated by either cAMP-dependent protein kinase or the AKT protein kinase homolog Sch9. We also demonstrate that Cdc34-S97 phosphorylation is elevated under conditions of active cell growth and division while it is decreased under conditions causing cell cycle arrest. These results provide a direct link between the growth regulating protein kinases PKA and Sch9 and early cell division events through post-translational modification of the ubiquitin-conjugating enzyme Cdc34.

## Results

### Genetic interactions among S73/S97/loop residues that define Cdc34/Ubc7-like class of E2s

**Figure 1:**
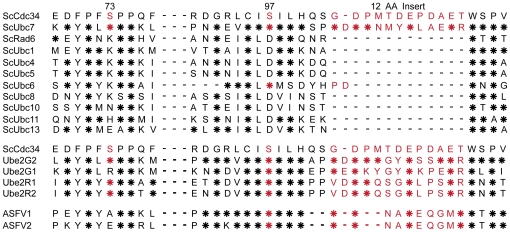
Alignment of E2s: The serine/serine/insert motif is conserved in all Cdc34 family members. (A) Partial alignment of yeast E2s and Cdc34/Ubc7 family members. Red indicates amino acid residues unique to the Cdc34 family of E2s (the regulatory triad). Asterisks represent identities to Cdc34. Dashes represent gaps. Sc sequences are from *S. cerevisiae*. Ube2 sequences are from humans. ASFV sequences are ASFV1 –African swine fever virus (GI:9628248) and ASFV2 –African swine fever virus (GI:450743).

A partial sequence alignment of the yeast ubiquitin conjugating enzymes and Cdc34 orthologs is shown in Figure 1. This partial alignment centers around two highly conserved regions of the E2 enzymes. S73 is centered within the highly conserved motif – [FY]P[FLY][ST]PP - while S97 is centered within the highly conserved motif - [LVI]C[IL]S[IMV][IL]. These two sequences are almost completely restricted to the E2 enzymes within yeast. The acidic loop region begins seven amino acid residues COOH-terminal to the active site cysteine at position 95 in Cdc34. As described above, there are robust genetic and functional relationships among S73, S97 and a twelve amino acid “loop” region (residues 103–114 of Cdc34) that surround the catalytic cysteine (C95). Ubc7, like Cdc34, contains serine residues at the positions equivalent to yeast Cdc34 S73 and S97. In contrast, the majority of E2s, of which Rad6 is a classic example, have a lysine residue and an aspartic acid residue, respectively, within these conserved regions and lack the “loop” region. This motif of serine/serine/insert or lysine/aspartic acid/no insert is conserved among most eukaryotic E2s [13].

**Figure 2:**
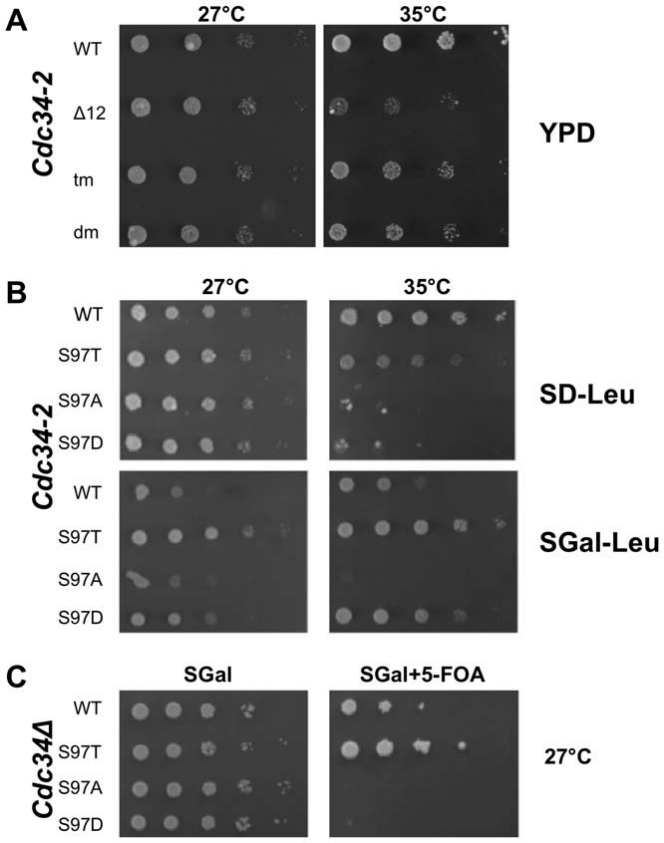
Complementation of *cdc34-2* and *cdc34Δ* strains by Cdc34 S97 Mutants. A and B) YL10-1, a *cdc34-2* temperature sensitive strain, bearing 2 µm plasmids encoding the indicated *CDC34* mutant under control of the *GAL10* promoter were spotted in tenfold serial dilutions on the indicated media and grown at the indicated temperatures for 3 days. C) YL18, a *cdc34Δ* strain harboring a *URA3* marked plasmid encoding wild type *CDC34* and a *LEU2*-marked plasmid encoding the indicated *CDC34* allele under control of the *GAL10* promoter were spotted in tenfold serial dilution on the indicated media and grown at 30°C for 3 days.

Previously, we and others determined that the Cdc34^S73K/S97D^ or Cdc34^S97D^ enzymes are non-functional both *in vivo* and *in vitro* and the Cdc34^Δ103–114^ mutant supports growth much more poorly than Cdc34 [11], [13]. Interestingly though, a mutant Cdc34 combining the S73K, S97D, and Δ103–114 (hereafter referred to as Cdc34^tm^) mutations is viable, even when introduced at the normal *CDC34* chromosomal locus under the *CDC34* promoter [11], [13]. In fact, Cdc34^S97D^ can be rescued by combining this mutation with the Δ103–114 mutation (hereafter referred to Cdc34^dm^; Fig. 2A). Thus there is a very strong genetic relationship between S97 and the loop region of Cdc34.

During the course of reproducing viability assays, we found that the *CDC34* ^S97D^ mutation can complement a *cdc34-2* temperature sensitive strain at a non-permissive temperature (Fig. 2B). Previous studies utilized 36°C as the non-permissive temperature and used patch assays during complementation experiments. By reducing the non-permissive temperature to 35°C and testing complementation by a spot dilution assay, several observations were made. *CDC34* ^S97D^ can complement a *cdc34-2* temperature sensitive strain at 35°C while *CDC34* ^S97A^ does not (Fig. 2B and C). However, neither *CDC34* ^S97D^ nor *CDC34* ^S97A^ complements *cdc34Δ*. Thus while neither *cdc34*-2 (*CDC34* ^G58R^) nor *CDC34* ^S97D^ retain Cdc34 function at 35°C, each can suppress the growth defect of the other. This intragenic suppression suggests that multiple Cdc34 proteins work together during ubiquitylation events. On the other hand, *CDC34* ^S97T^, a mutation that ostensibly permits phosphorylation at this residue, complements both the *cdc34-2* temperature sensitive and *cdc34Δ* strains in spot dilution assays (Fig. 2). The viability of *CDC34* ^S97T^, coupled with the previous result that Cdc34 can be found phosphorylated *in vivo* [16], provided the rationale for the experiments described below to determine whether Cdc34-S97 is a site of phosphorylation. Intriguingly, these results also demonstrate that over-expression of *CDC34* on galactose medium is growth inhibitory (Fig. 2B and C).

### Cdc34 is phosphorylated *in vivo* on S97

**Figure 3:**
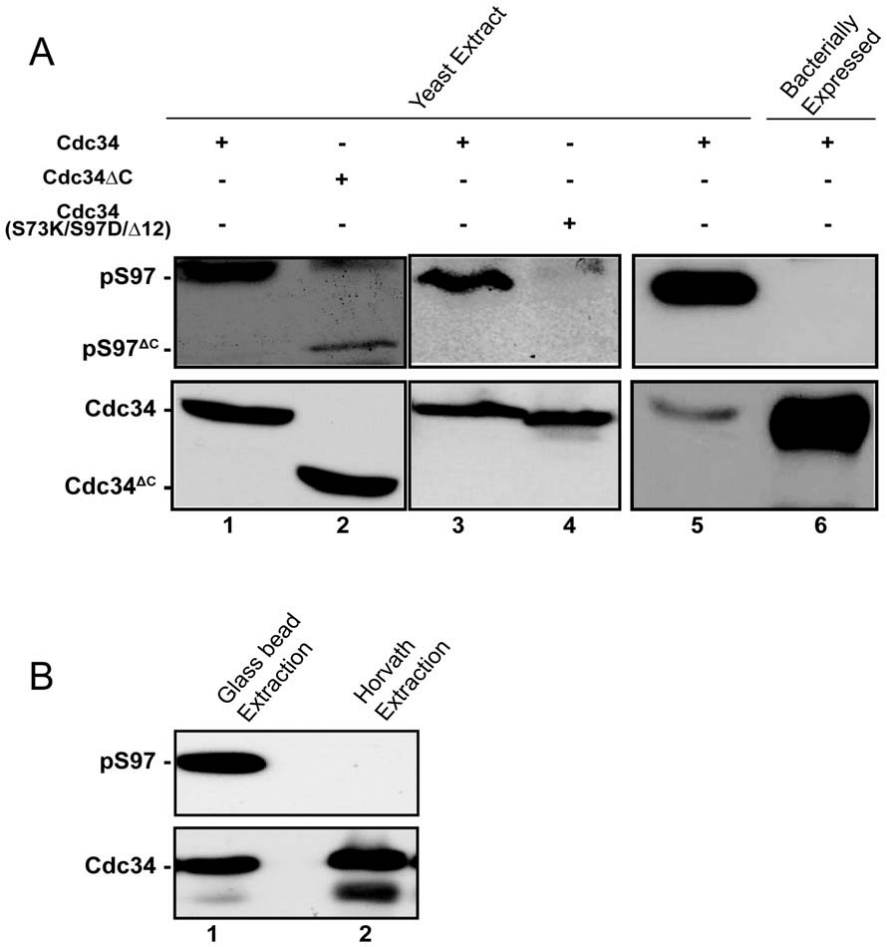
Cdc34 S97 is phosphorylated in vivo and its detection is prohibited by certain protein extraction conditions. A) Soluble protein from yeast cells, expressing from the *CDC34* chromosomal locus, either wild type Cdc34 (lanes 1, 3 and 5), a COOH-terminally truncated Cdc34 encoding amino acids 1–244 (lane 2) or a Cdc34^tm^ mutant encoding the S97D mutation (lane 4) or bacterially expressed Cdc34^6XHis^ (lane 6) were analyzed by western blot using the α-pS97 phospho-specific or α-Cdc34 antibodies. B) An equal amount of soluble yeast protein extract made by either the glass bead or Horvath extraction protocol (see Materials and Methods) was analyzed by western blot using the α-pS97 phospho-specific and α-Cdc34 antibodies.

Cdc34 is phosphorylated *in vivo* on serine residues [16] and several of the serine phosphorylation sites have been mapped [17], [18]. Due to the fact that *CDC34* ^S97T^ is functional both *in vivo* and *in vitro* and that Cdc34-S97 is highly conserved across eukaryotic species, we hypothesized that Cdc34-S97 is a site of phosphorylation. To test this hypothesis, we generated antibodies that specifically recognize Cdc34 phosphorylated at S97 (S97p). This antibody, termed α-pS97, recognizes a protein from whole cell yeast lysate which migrates at the same position as Cdc34 (Fig. 3A, lane 1). When full length Cdc34 is replaced by a COOH-terminally truncated Cdc34 that migrates noticeably faster through an SDS-PAGE gel, the α-pS97 antibody recognizes a protein which migrates at the same location as COOH-terminally truncated Cdc34 (Fig. 3A, lane 2) indicating that the α-pS97 antibody recognizes Cdc34 and that Cdc34 is the only protein contributing to the signal at the approximate molecular mass of 42 kDa. The α-pS97 antibody does not recognize Cdc34^tm^ (Fig. 3A, lane 4) indicating that this antibody is specific to S97. Moreover, even when loaded in excess of endogenous yeast Cdc34, bacterially expressed Cdc34, which is not phosphorylated, is not recognized by α-pS97 (Fig. 3A, lane 6). From these studies, we conclude that the α-pS97 antibody is specific for Cdc34-S97p and that Cdc34 is phosphorylated *in vivo* on S97. Furthermore, these data indicate that the COOH-terminus of Cdc34 (amino acids 245–295) is an important determinant of Cdc34-S97 phosphorylation, since the level of Cdc34-S97 phosphorylation is dramatically reduced on COOH-truncated Cdc34 (Fig. 3A, lane 2).

An important methodological consideration for these experiments is that Cdc34-S97 phosphorylation becomes undetectable when the protein extract is made by the Horvath-Riezman method [19], a common method for yeast cell protein extraction which requires heating the sample in SDS for 5 minutes (Fig. 3B, lane 2). However, cell disruption by the glass bead method in either the breaking buffer described in materials and methods or 8 M urea allows S97 phosphorylation to be detected by western blotting.

### Phosphorylation of S97 is induced in the G1 phase

**Figure 4:**
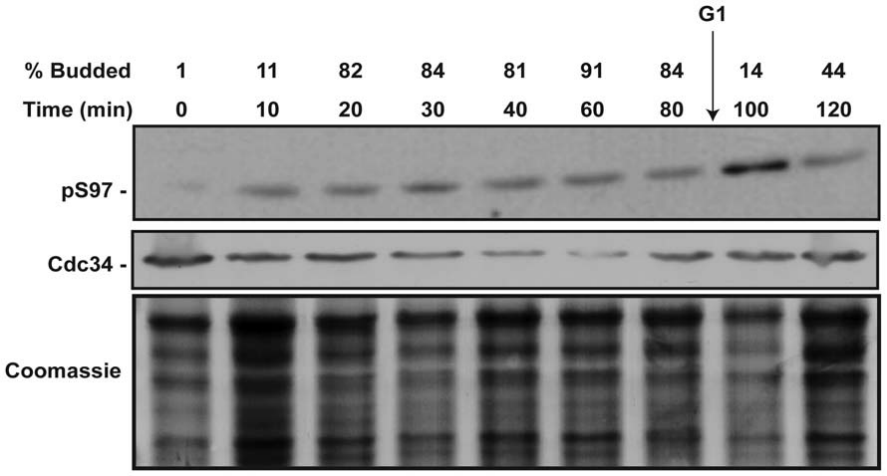
Cdc34 S97 phosphorylation increases in the G1 phase of the cell cycle. Strain RC150 was grown to mid-log phase and arrested with a final concentration of 0.5 µg/ml µM of α-factor. After 3 hours, >95% of the cells had arrested with a shmoo-like morphology. Cells were washed and resuspended in fresh YPD. Cells were collected at the indicated time points and total soluble protein was analyzed by western blot using the α-pS97 phospho-specific and α-Cdc34 antibody. At least 100 cells were counted per time point to determine the budding index.

Cdc34 in conjunction with SCF complexes has a plethora of substrates which are targeted for proteasome mediated degradation. The known substrates whose degradation is required for timely cell cycle progression are all ubiquitylated and degraded in the G1 phase of the cell cycle. Thus, we hypothesized that Cdc34-S97 phosphorylation is regulated through the cell cycle. To test this hypothesis, cells were synchronized in late G1 with α-factor. After three hours, >95% of the cells were arrested with a mating projection, or shmoo, morphology. The α-factor was washed out; cells were resuspended in fresh medium; and cells were then collected at the indicated times (Fig. 4). At the initial removal of α-factor, Cdc34-S97 phosphorylation is very low, but quickly rises to a level that remains constant through most of the cell cycle until the second G1 phase is reached. At the second G1 phase the level of Cdc34-S97 phosphorylation increases dramatically (Fig. 4). α-factor arrests cells late in G1, just prior to the initiation of DNA replication and bud emergence. When incubated at a non-permissive temperature, cells harboring temperature sensitive alleles of *cdc28* or *cdc25* arrest at a point (or points) prior to α-factor induced arrest and remain sensitive to α-factor when returned to a permissive temperature [20], [21]. The fact that induction of S97 phosphorylation does not occur immediately after α-factor release and only after a full cell division cycle suggests that the S97 phosphorylation increase occurs early in G1, prior to the α-factor arrest point. This finding is consistent with Cdc34-S97 phosphorylation occurring on active Cdc34 when its cell cycle substrates are degraded.

### Identification of kinases that may phosphorylate Cdc34-S97

**Figure 5:**
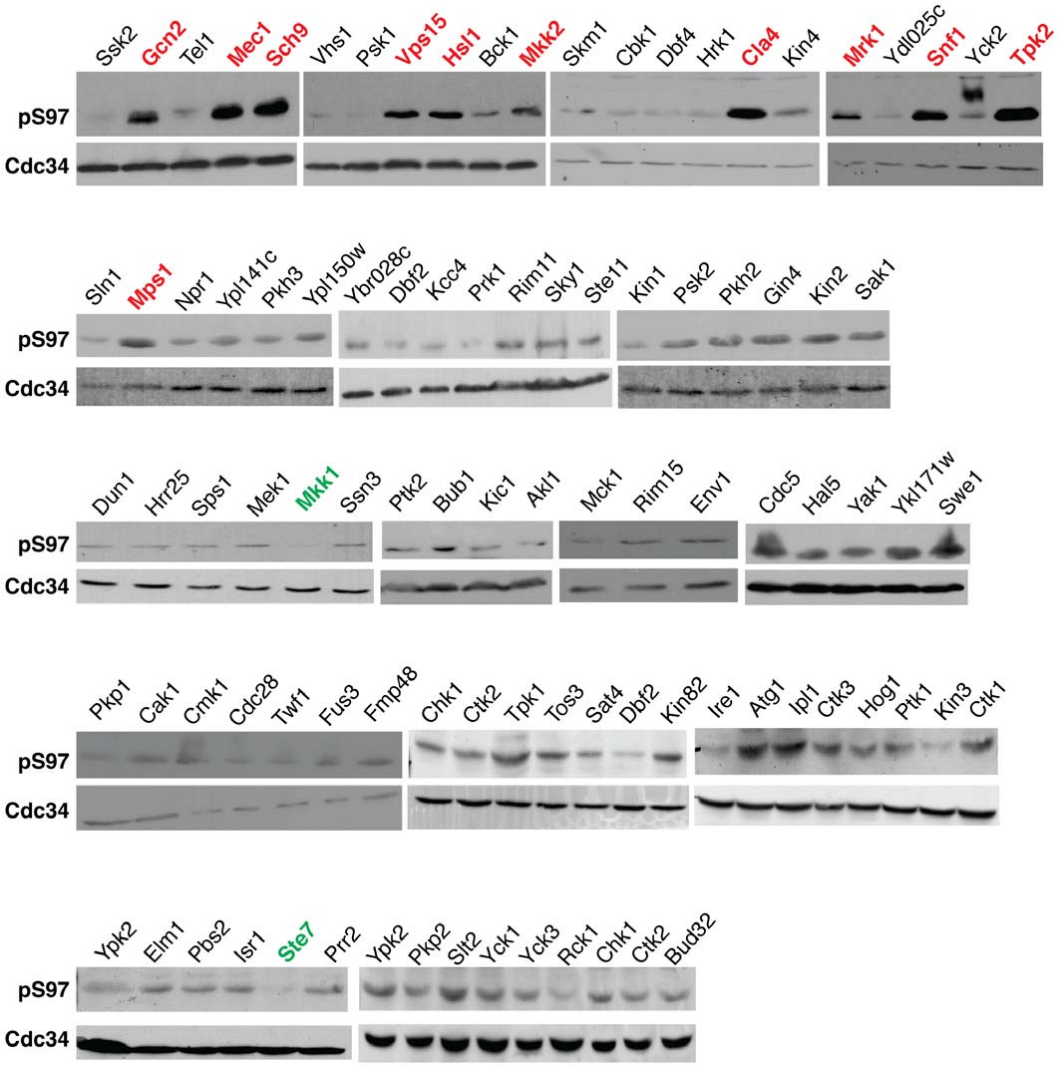
A screen for kinases whose overexpression alters the level of Cdc34-S97 phosphorylation. A) Total soluble protein from an array of strains each overexpressing the indicated kinase was analyzed by western blot using the α-pS97 phospho-specific and α-Cdc34 antibodies.

Upon establishing that Cdc34 is phosphoryIated on S97 in a cell cycle dependent manner, we focused on identifying the protein kinase(s) responsible for this event. We began our screen for candidate Cdc34-S97 kinases by an in vivo over-expression assay. This was done to avoid overlooking candidate protein kinase genes that are essential as would have been the case if we had begun our analysis with a collection of yeast containing viable protein kinase deletion mutants. To identify the kinase(s) responsible for phosphorylating Cdc34-S97, we utilized a genomic ordered array of 124 yeast strains, each expressing a unique GST-kinase fusion under the control of a copper inducible promoter. Construction and utilization of the yeast ORF collection has been described previously [20]. Strains were grown to mid exponential phase in minimal media and GST-kinase expression was induced with the addition of copper sulfate. In total, more than 100 unique kinases were screened for their effect on Cdc34-S97 phosphorylation in this manner (Fig. 5). Using this assay, a number of kinases (Tpk2, Sch9, Vps15, Mkk2, Cla4, Mrk1, Mec1, Mps1, Snf1 and Gcn2), when over-expressed, caused the greatest increase in the level of Cdc34-S97 phosphorylation *in vivo*. There are also two MAP kinase kinases, Mkk1 and Ste7, which when over-expressed noticeably reduce the level of Cdc34-S97 phosphorylation (Fig. 5). While we considered this to be a comprehensive screen of the yeast kinome, it did not include the full complement of kinase activity in yeast. Several kinase encoding genes were not present in our array including *VPS34*, *TOR1*, *TOR2* and *STE20*. Furthermore, a number of strains did not grow to sufficient density for analysis of Cdc34-S97 phosphorylation. We also realize that other kinases require targeting subunits and that without simultaneous over-expression of both the catalytic and regulatory subunits, the activity of these kinases against certain substrates will remain unchanged. Interestingly though, most of the candidate kinases for Cdc34-S97 phosphorylation are known regulators of nutritional status and growth control.

**Figure 6:**
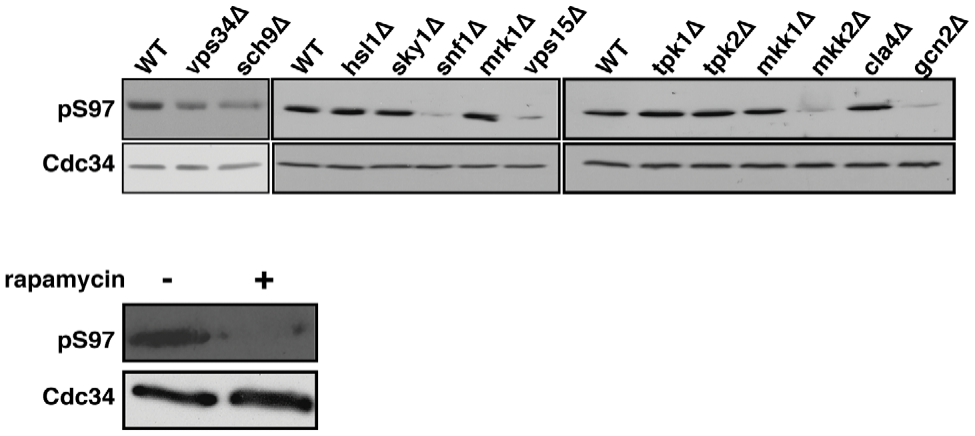
Loss of Vps34, Vps15, Sch9 and Torc1 activity reduce Cdc34 S97 phosphorylation. Total soluble protein from strains lacking the indicated kinase or from a strain treated with 200 nM rapamycin for 1 hour was analyzed by western blot using the α-pS97 phospho-specific and α-Cdc34 antibodies.

To extend the investigation of kinases affecting Cdc34-S97 phosphorylation, we analyzed the level of Cdc34-S97 phosphorylation in strains lacking the individual kinases whose over-expression causes an increase in Cdc34-S97 phosphorylation. This analysis did not include strains lacking *MEC1* or *MPS1* as these genes are essential. Based on this analysis, the level of S97 is reduced in strains lacking Gcn2, Mkk2, Sch9, Snf1, and Vps15/Vps34 (Fig. 6). We also examined the effect of a *VPS34* deletion on Cdc34-S97 phosphorylation. *VPS34* was not included in our set of protein kinase overexpressing strains; however, Vps34 and Vps15 function as a complex [21]. Ultimately, loss of either Vps15 or Vps34 results in a reduction in Cdc34-S97 phosphorylation (Fig. 6).

Finally, the absence of a number of these protein kinases (Tpk1, Tpk2, Mrk1, Hsl1, Cla4, or Mkk1) does not noticeably reduce the level of S97 phosphorylation (Fig. 6). While there are multiple reasons that might explain why the loss of these kinases does not reduce the level of S97 phosphorylation, one likely explanation is that each of these remaining protein kinases is a member of a structurally related subfamily of protein kinases whose members are thought to have overlapping functions (the cAMP-dependent protein kinases-Tpk1/Tpk2/Tpk3, the Gsk3-like protein kinases-Mrk1/Rim11/Mck1/Ygk3, the PAK kinases-Cla4/Ste20/Skm1, and the Hsl1/Kcc4/Gin4 family). Additionally, we cannot eliminate the possibility that a particular kinase might only influence the phosphorylation of a small pool of Cdc34 whose reduced phosphorylation is undetectable when the entire pool of Cdc34-S97p is probed. Cdc34 associates with many SCF complexes which are unique in their F-box component and it is also possible that different kinases might influence the phosphorylation of Cdc34-S97 only in the context of specific SCFs.

For several reasons, we also chose to examine specifically whether the Tor kinases controlled Cdc34-S97 phosphorylation for several reasons. First, as shown above, several other nutrient sensing kinases alter Cdc34-S97 phosphorylation. A function of the TORC1 complex (Tor1, Kog1, Lst8, and Tco89) is as a sensor of nitrogen availability in eukaryotic cells. Secondly, both Vps15, a kinase which activates TORC1, and Sch9, a kinase activated by TORC1, increase Cdc34-S97 phosphorylation when over-expressed and strains lacking either of these kinases have reduced Cdc34-S97 phosphorylation. Thus TORC1 could be part of a Vps15/Vps34-TORC1-Sch9 cascade leading to Cdc34-S97 phosphorylation. Finally, neither Tor1 nor Tor2 are part of the collection of copper inducible kinases used in the initial screen; however, Tor activity can be inhibited by the natural compound rapamycin. As predicted, Cdc34-S97 phosphorylation is reduced when cells are treated with the Tor kinase inhibitor rapamycin (Fig. 6).

### Altered PKA activity affects S97 phosphorylation

**Figure 7:**
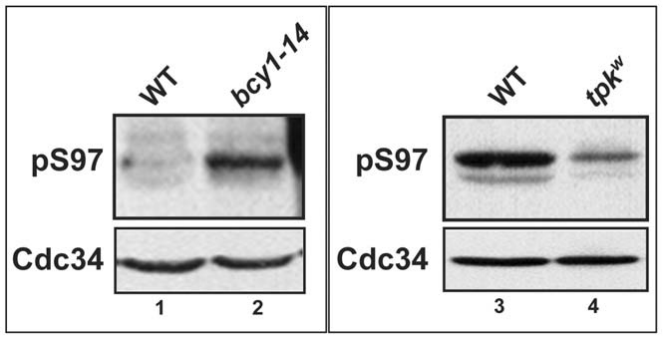
The cAMP/Protein Kinase A pathway regulates Cdc34 S97 phosphorylation. A) Total soluble protein from the strains KT945 (WT, lanes 1 and 3), RS13-58A-1 (*tpk1* ^w^, lane 2) and KT1126 (*bcy1*–14, lane 4) was analyzed by western blot using α-pS97 phospho-specific and α-Cdc34 antibodies.

An upstream component of the cAMP/PKA pathway, *RAS2*, genetically interacts with *CDC34* [22]. Compromising the activity of protein kinase A, either by deleting *RAS2* or by growing cells on a non-fermentable carbon source which reduces intracellular cAMP levels, inhibits the growth of *cdc34-2* temperature sensitive strains at otherwise permissive temperatures [22]. These findings suggest that Cdc34 and PKA act in a common pathway and together with the fact that Cdc34-S97 phosphorylation increases when *TPK2* is overexpressed led us to test whether S97 phosphorylation levels are affected in other circumstances where PKA activity is altered. There are three isoforms of the catalytic subunit of PKA, Tpk1–3 (reviewed in [23]). Bcy1 is the regulatory subunit which inhibits PKA catalytic activity in the absence of cAMP. Cyclic AMP binds Bcy1 causing its release from the catalytic subunits, resulting in increased PKA activity [24]. Strains lacking *BCY1* have increased PKA activity and are sensitive to heat shock and nutrient deprivation due specifically to increased PKA activity [25]. We find that the level of Cdc34-S97 phosphorylation is increased in cells harboring the crippled *bcy1–14* allele (Fig. 7, lane 2), consistent with the conclusion that PKA controls Cdc34-S97 phosphorylation.

Strains lacking all three *TPK* genes are inviable [26] and strains lacking either *TPK1* or *TPK2* do not have altered levels of Cdc34-S97 phosphorylation (Fig. 6). Therefore, we utilized a strain lacking both *TPK2 and TPK3 genes* and harboring a weak TPK1 allele, termed *tpk1^w^*, whose protein product shows reduced cAMP-dependent protein kinase activity [27]. We find that Cdc34-S97 phosphorylation is reduced in this strain lacking *TPK2* and *TPK3* and harboring the *tpk1^w^* allele (Fig. 7, lane 4). In all, these experiments demonstrate that during exponential growth in glucose the level of Cdc34-S97 phosphorylation can be controlled by protein kinase A.

### Reconstitution of Cdc34-S97 phosphorylation in vitro

**Figure 8:**
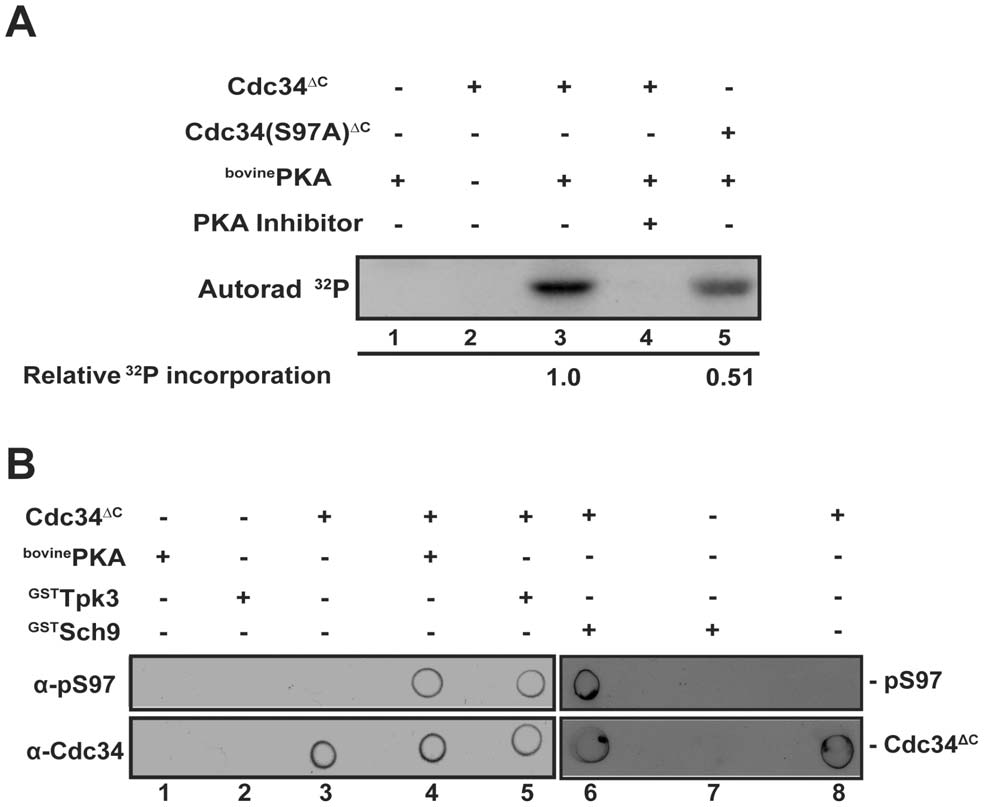
PKA directly phosphorylates Cdc34 on S97. A) Cdc34ΔC^6XHis^ and Cdc34(S97A)ΔC^6XHis^ were phosphorylated *in vitro* by addition of bovine PKA and [γ-^32^P]ATP. Protein kinase A inhibitor (Sigma Aldrich, USA) was added to the indicated reactions. The reaction components were separated by SDS-PAGE and autoradiographed. To quantitate the relative amount of ^32^P incorporation, Cdc34 was excised from the dried and analyzed by liquid scintillation counter. B) Cdc34ΔC^6XHis^ was phosphorylated *in vitro* by either bovine PKA, ^GST^Tpk3 or ^GST^Sch9. The reaction was spotted onto a PVDF membrane and probed with either α-pS97 phospho-specific or α-Cdc34 antibody.

While genetic and physiological manipulation of Cdc34-S97 phosphorylation provides compelling evidence that specific protein kinases can control this event, these experiments do not allow us to distinguish between a direct Cdc34-S97 phosphorylating activity and indirect effects. Therefore *in vitro* phosphorylation assays were performed to determine which protein kinase(s) implicated in Cdc34-S97 phosphorylation directly phosphorylate Cdc34 on S97. Protein Kinase A has a strong preference for arginine residues at positions −2 and −3 and a preference for an amino acid residue with a hydrophobic side chain at the +1 position relative to the phosphorylation site [28], [29], [30]. A second consensus sequence for PKA has been identified as R_−6_-X-X-R_−3_-X-X-(S/T_0_)-B_+1_ with X referring to any amino acid residue and B referring to an amino acid residue with a hydrophobic side chain [31]. While Cdc34-S97 is not part of a PKA consensus sequence, Cdc34 has arginine residues at positions −4 and −7 and an isoleucine residue at the +1 position relative to S97. Therefore we tested whether PKA is able to phosphorylate Cdc34-S97 *in vitro*. Indeed, Cdc34 can be phosphorylated by bovine PKA while the Cdc34^S97A^ mutant is phosphorylated to a lesser degree (Fig. 8A). Cdc34 phosphorylation is completely abolished by addition of Protein Kinase A Inhibitor to the reaction (Fig. 8A). To confirm these findings, we probed the *in vitro* phosphorylation reaction products with the α-pS97 antibody which shows that both bovine PKA and ^GST^Tpk3, purified from yeast, are able to phosphorylate Cdc34-S97 (Fig. 8B). Furthermore, ^GST^Sch9 purified from yeast is also able to phosphorylate Cdc34 on S97 *in vitro* in similar assays (Fig. 8B). The Sch9/Akt family members also modify an arginine-directed consensus sequence (for review, see [32]). Thus we conclude that Protein Kinase A and Sch9, both AGC family protein kinases, directly phosphorylate Cdc34-S97 *in vitro* and support our findings that alterations of these protein kinases dramatically affect S97 phosphorylation *in vivo*.

### The S73/S97/Loop Motif is Critical for Chronological Lifespan and rapamycin resistance

**Figure 9:**
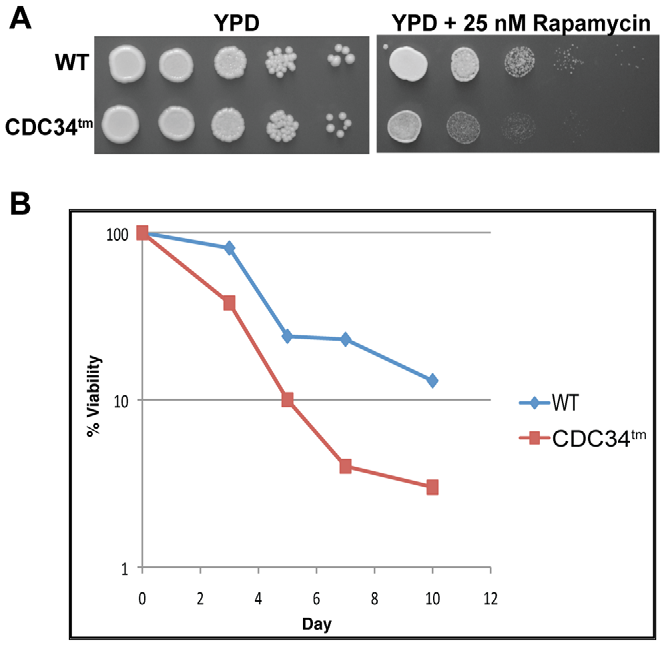
The Cdc34 S73/S97/loop motif increases chronological lifespan and is required for rapamycin resistance. A) Prototrophic strains BL2 (WT) and RRC85 (CDC34^tm^) were grown in synthetic defined liquid media lacking all amino acids for the indicated number of days at 32 C and the percent viability was determined by spotting a fraction of the culture on a YPD plate and counting colonies after incubation for 3 days at 32 C. B) Strains BL2 and RRC85 were grown overnight in SD lacking all amino acids, diluted to 5×10^7^ cells/ml and spotted in ten-fold serial dilution on YPD and YPD+25 nM rapamycin plates. Plates were incubated at 32°C for 3 days and then photographed.

Conditions, such as nutrient depletion or exposure to mating pheromone, which require timely cell cycle arrest in the G1 phase, depend on destabilization of the G1 cyclins Cln1, Cln2 and Cln3 and stabilization of the cyclin dependent kinase inhibitors Sic1 and Far1. Overexpression of *CLN2* or loss of *FAR1* prevents mating pheromone induced cell cycle arrest [33], [34]. And, as mentioned above, ectopic *CLN3* expression or loss of *SIC1* compromises G1 arrest and ultimately the capability of a yeast cell to withstand prolonged periods of nutrient depletion [35], [36]. Furthermore, over-expression of *CLN3* or loss of *SIC1* makes yeast sensitive to otherwise tolerable levels of the TORC1 inhibitor rapamycin, which mimics nutrient deprivation [36]. Cdc34^tm^ increases the rate of Sic1 degradation while decreasing the rate of Cln1 degradation [15]. Therefore, we hypothesized that the highly conserved S73/S97/loop motif of Cdc34 contributes to the ability of cells to survive low dose rapamycin treatment or nutrient depletion. Indeed, we find that a normally permissive level of rapamycin inhibits growth of *CDC34^tm^* cells (Fig. 9A) likely due to improper regulation of Sic1, Cln1 and Cln2.

Aging studies of *S. cerevisiae* are classically conducted by two methods. The first method allows for the determination of replicative lifespan which is a study of mitotically dividing cells. An individual yeast cell will undergo a finite number of divisions before dying. There is a degree of strain specific variation in the number of divisions a new mother can undergo prior to senescence but, on average, a single yeast cell can give rise to ∼20–25 daughters (reviewed in [37]). Determination of the viability of non-dividing yeast cells in the G_0_ or post-diauxic state, termed chronological lifespan, is another method by which aging is assessed in *S. cerevisiae*. After approximately three days in synthetically defined media, yeast cells stop dividing and significantly slow their metabolic activity. Nutrients become limiting under these conditions but the cells are not starving and it has been postulated that this state resembles in some respects the conditions of post-mitotic cells in higher organisms [38].

To test survival of *CDC34^tm^* yeast during a prolonged period of nutrient depletion, isogenic wild type and *CDC34^tm^* strains were grown to stationary phase in liquid culture and the number of individual cells capable of forming a colony was determined over a period of ten days. After 48 hours in defined liquid media, the cells have ceased dividing and some of the essential nutrients have been depleted [39]. As Figure 9 shows, *CDC34* ^tm^ cells are not initially compromised for survival (Fig. 9B, day 0) but as the quiescent state is extended, *CDC34* ^tm^ yeast are significantly less robust; and by day 10 their survival rate is more than fivefold lower than the WT strain (Fig. 9A, days 3–10). This result explicitly demonstrates the selective pressure on the yeast *S. cerevisiae* to retain Cdc34 rather than Cdc34^tm^. In nature, nutrient limitation is commonly encountered. Thus, genetic elements, like the S73/S97/loop motif, which contribute to survival during nutrient limitation, are highly desirable.

## Discussion

Regulation of SCF-dependent UPS events occurs through substrate level phosphorylation as in the case of the SCF [6], [7], [8]. In other cases, E3 modifications such as phosphorylation and neddylation are key regulators of ubiquitylation (for review, see [40], [41]). F-Box protein stability within the SCFs has also been shown to be a viable mechanism for controlling ubiquitylation events (for review, see [42]). More recently, potential regulation at the level of the E2 has been described. Cdc34 function can be modulated in a manner dependent on COOH-terminal phosphorylation by casein kinase 2 [14], [17], [18], [43] most likely by preventing the formation of unattached ubiquitin chains. Rad6 and has also been shown to be regulated by phosphorylation [44].

By screening nearly the entire set of yeast kinases, we have discovered several kinases that affect Cdc34-S97 phosphorylation. In looking for relationships among the kinases that increase Cdc34-S97 phosphorylation it became apparent that many are responsive to intracellular or extracellular nutrient conditions and in turn, loss of these signaling pathways results in G1 arrest. Gcn2, PKA, Sch9, Snf1, Tor, and Vps34/Vps15 all have a role in nutrient sensing and the resulting intracellular adaptations to the nutritional environment. PKA is activated by glucose through an increase in intracellular cAMP [45]. The Targets of Rapamycin, Tor1 and Tor2, are also responsive to nutrients and are active under conditions of adequate nitrogen and carbon. Inhibition of Tor1 kinase activity by rapamycin mimics the effects of nitrogen depletion. In mammalian cells, and possibly yeast cells as well, the Vps34/Vps15 complex is activated by amino acids, while glucose starved cells have no detectable Vps34 activity [46], [47].

Gcn2 and Snf1, on the other hand, are most well characterized for their functions when nutrient conditions are less favorable. Gcn2 becomes active when yeast cells lack external sources of amino acids (for review, see [48]). Uncharged tRNAs accumulate upon amino acid starvation. The Gcn2 histidyl-tRNA binding domain binds the uncharged tRNAs and becomes active. This allows Gcn2 to phosphorylate eIF2α, in turn slowing the rate of general translation but increasing the rate of Gcn4 translation. Gcn4 is a transcriptional activator which induces the amino acid biosynthetic genes. Snf1 is a yeast homolog of AMP Kinase. It is activated by a high AMP/ATP ratio but, unlike mammalian AMPK, is not directly allosterically activated by AMP [49]. Growth on low glucose or non-preferred carbon sources lead to high AMP/ATP ratios and therefore strains lacking *SNF1* struggle to grow on sucrose, galactose, acetate and ethanol. This is due to the fact that Snf1 has a major role in activation of glucose-repressed genes such as genes encoding the gluconeogenic enzymes. Thus a common feature of Gcn2 and Snf1 is that both are necessary for cells to undergo a metabolic shift from preferred to non-preferred conditions - from importing amino acids to synthesizing amino acids in the case of Gcn2 and from fermentative to respiratory growth in the case of Snf1.

As nutrient sensing is an integral function of the G1 phase, it is not surprising that loss of nutrient sensing kinases can result in an arrest in the G1 phase. For example, strains lacking all the PKA isoforms or TOR arrest in G1. Consistent with their established roles in nutrient sensing during the G1 phase, there are inter-relationships among the kinases that control Cdc34-S97 phosphorylation. The primary example is phosphorylation of Sch9 by both Snf1 and the TORC1 complex [50], [51]. Phosphorylation of Sch9 on its COOH-terminus by TORC1 increases Sch9 activity against the ribosomal S6 subunit, suggesting that Sch9 functions as the S6 kinase in yeast [50]. Furthermore, the Vps15/Vps34 heterodimer activates TORC1 by an unknown mechanism [47]. From these studies, it appears that Vps34/Vps15 activates Tor, in turn activating Sch9, which is consistent with the finding that each of these kinases controls Cdc34-S97 phosphorylation. Snf1 also directly phosphorylates and likely activates Sch9. This phosphorylation occurs in older cells of a population and appears to promote yeast cell senescence [51].

Protein Kinase A is inactive when bound to the cAMP responsive protein, Bcy1. The signaling molecule cAMP binds to Bcy1 and allows the PKA catalytic subunits to dissociate and become active. The kinases Mrk1 and Mkk2, which increase S97 phosphorylation when overexpressed, negatively regulate Bcy1 cytoplasmic accumulation. Regulation of Bcy1 by Mrk1, its GSK-3 orthologs and Mkk2 has only been observed under conditions of heat stress (growth at 37°C) [52]. One possibility is that overexpression of *MRK1* or *MKK2* may cause re-localization of Bcy1 and result in increased Tpk1–3 activity towards Cdc34-S97. Mec1 activates PKA under conditions of DNA damage [53] which offers a possible explanation for the increase in Cdc34-S97 phosphorylation in a strain overexpressing *MEC1*. Since *MEC1* is an essential gene, it is difficult to assess whether Mec1 contributes to PKA activation under steady state growth conditions.

Sch9 and the PKA catalytic subunits (Tpk1–3) are members of the AGC (Protein Kinase A/G/C) kinase family which consists of the cAMP dependent protein kinases, the cGMP dependent protein kinases and Protein Kinase C. Sch9 and PKA may function in redundant pathways as suggested by the findings that *SCH9* acts as a multi-copy suppressor of PKA signaling defects and activation of PKA eliminates the slow growth defects of *sch9Δ* cells [54]. Furthermore, Sch9 and PKA function redundantly to inhibit autophagy in rich media [55]. AGC family kinases have similar consensus sequences neighboring the amino acid to be phosphorylated. Most frequently, two or three basic amino acids are found N-terminal to the serine or threonine which accepts the phosphate group. Protein Kinase A was screened against a peptide substrate library and was found to have a strong preference for arginine amino acid residues at positions −2 and −3 and a preference for an amino acid with a hydrophobic side chain at the +1 position [28],[29],[30]. A second consensus site for PKA has been identified as R_−6_-X-X-R_−3_-X-X-(S/T_0_)-B_+1_ with X referring to any amino acid residue and B referring to an amino acid residue with a hydrophobic side chain [31]. We find that Cdc34-S97 is a substrate for PKA and, although atypical, Cdc34-S97 is part of a reasonable AGC-type sequence with arginine residues at positions −4 and −7 and isoleucine at +1 relative to S97.

**Figure 10:**
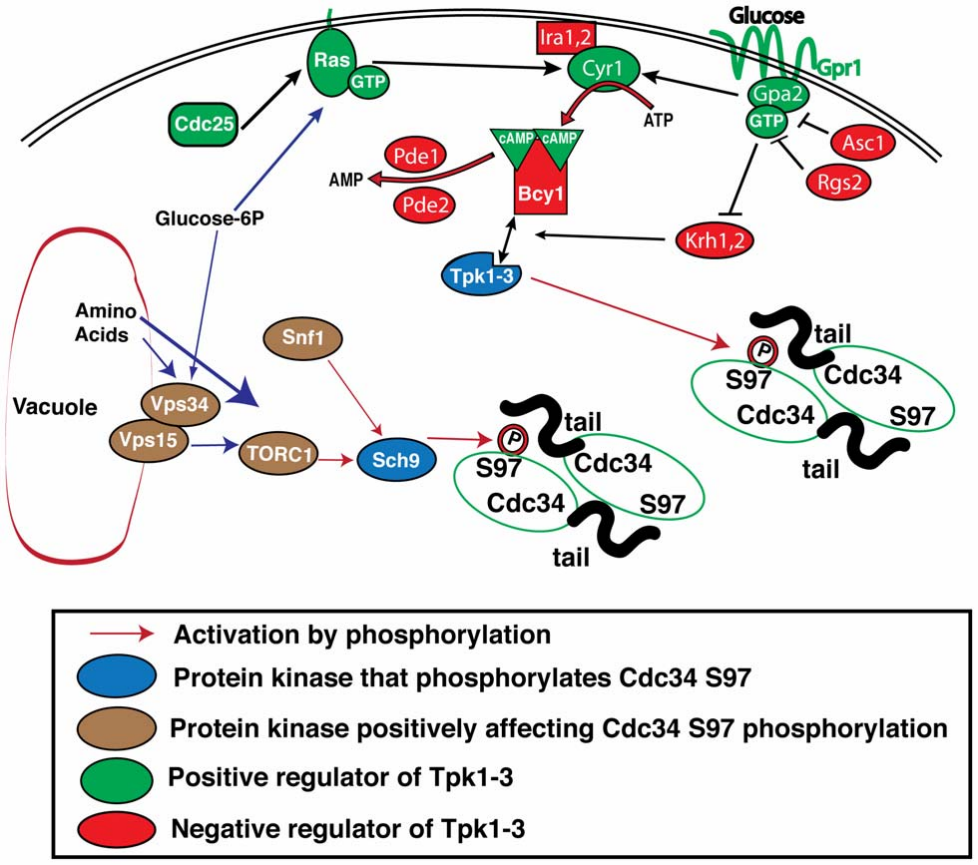
Model of Cdc34 S97 phosphorylation.

Although we have not yet tested the impact of S97 phosphorylation on Cdc34 ubiquitin conjugating activity, genetic and molecular results suggest that Cdc34-S97 phosphorylation activates the enzyme. Mutating S97 to alanine or aspartic acid inhibits the *in vivo* and *in vitro* activity of Cdc34; however, an S97T mutation is functional. There are published genetic interactions between the cAMP/Ras/PKA pathway and Cdc34 suggesting that PKA stimulates Cdc34 activity [22]. Furthermore, elimination of kinases that are positive effectors of Cdc34-S97 phosphorylation, appear to reduce Cdc34 activity *in vivo*. We hypothesize that Cdc34-S97 phosphorylation increases the ubiquitin conjugating activity of Cdc34. The mechanism is unclear but one possible working model is as follows. Self-association is required for Cdc34 function and Cdc34^S97D^ mutants do not self-associate [11], [13]. However, fusion of an artificial dimerization domain, such as GST, to the Cdc34^S97D^ mutant restores its ability to polyubiquitylate a natural substrate [10]. The Cdc34^S97D^ can interact with an SCF complex [11] as demonstrated by the *in vivo* immunoprecipitation of ^Flag^Cdc34^S97D^ showing that it co-precipitates Cdc53. Wild-type Cdc34 enzyme was present in this assay and it is possible that wild-type Cdc34 dimerizes with Cdc34^S97D^, allowing Cdc34^S97D^ to interact with SCF. Our discovery that the Cdc34^S97D^ mutant can complement a *cdc34* temperature sensitive strain but not a null strain suggests that the residual activity of the temperature sensitive enzyme is supplying a component of the necessary activity to the Cdc34^S97D^ mutant which the Cdc34^S97A^ mutant cannot utilize. In light of the above discussion, it is possible that the Cdc34^S97D^ mutant and wild type enzymes form functional heterodimers and supply the necessary Cdc34 activity to the cell. In a physiological setting, this model could be extended to a scenario where two Cdc34 molecules dimerize after one has been phosphorylated on S97 (Fig. 10). Potentially these similar Cdc34 molecules, which are differentially modified at S97, have different activities, with one Cdc34 molecule conjugating the initial ubiquitin to the substrate and the other extending the polyubiquitin chain. Such a Cdc34 heterodimer may function by mimicking the E2 handoff mechanism proposed by Wu et al. [56] to explain the synergy between UbcH5 and Cdc34 in vitro.

The process of aging is extremely complex, influenced by diet and lifestyle. However, single gene mutations have been found in all model organisms including yeast, fruit flies, worms and mice which dramatically extend lifespan (for review see [39]). Evidence for a conserved mechanism of aging can be derived from the fact that mutations in genes including Sch9/AKT affect the lifespan of all model organisms mentioned above. Sch9 is 49% identical to human, fly and worm AKT over a stretch of ∼300 amino acids which encode the kinase domain. In these and other higher eukaryotes, AKT is activated by insulin and the insulin-like growth factor (IGF-1). Weak mutations in the IGF-1 pathway can extend the lifespan of worms more than two fold and that of fruit flies by 85% [57], [58], [59], [60]. Furthermore, Prop-1 (−/−) and Pit-1 (−/−) dwarf mice have decreased levels of IGF-1 and live ∼65% longer than wild type mice [61], [62], [63]. The aging phenotype of Cdc34^tm^ cells is consistent with Cdc34^tm^ cells becoming at least partially independent of PKA or Sch9 activity and no longer responding properly to growth arrest signals normally associated with the inactivation of these protein kinases, much like hyperactivation of the PKA pathway by loss of *IRA1* or hyperactivation of Ras [64].

## Materials and Methods

### Site directed mutagenesis

All site directed mutagenesis was carried out using the QuikChange II XL Site-Directed Mutagenesis Kit (Stratagene, California, USA) and associated protocol. Complementary forward and reverse primers, approximately 25 nucleotides in length, encoding the desired mutation(s) were used to amplify the appropriate plasmid. Following PCR amplification, 1 µl of *Dpn* I restriction enzyme was added to the reaction mix to digest the parental, methylated plasmid DNA and the reactions were incubated at 37°C for 1 hour. XL10-Gold Ultra-competent cells were transformed with two µl of *Dpn* I-treated DNA. Transformants were selected on LB+Ampicillin plates. Multiple clones from each transformation were verified by sequencing. The plasmids used in this work can be found in Table 1.

**Table 1:** Plasmids used in this study.

Plasmid Name	Vector	Yeast Marker	Bacterial Marker	Insert Gene	Source
pYL150	pSJ101	LEU2	ampicillin	CDC34	[13]
pYL029	pSJ101	LEU2	ampicillin	CDC34S73K/S97D/Δ103–114	[13]
pSJ101	pSJ101	LEU2	ampicillin		S. Johnson
pYL027	pSJ101	LEU2	ampicillin	CDC34insert delta	Yun Liu
pRC001	pSJ101	LEU2	ampicillin	CDC34S97D/delta	this study
pAG25	pFA6	natMX4	ampicillin	Nourseothricin N-acetyltransferase	EUROSCARF
AD002	pET21	none	ampicillin	CDC34deltaC(1–244)-6XHis	D. Skowyra
pRC004	pET21	none	ampicillin	CDC34deltaC(1–244 (S97A)-6XHis	this study

### Yeast growth media and genetic techniques

Standard rich (YPD) and defined minimal (SD) media were prepared as described previously. To transform yeast with plasmid DNA, a quick method was utilized. Briefly, 0.5 µg of the plasmid DNA, 20 µl of 2 mg/ml carrier DNA and four volumes of PEG/Li-acetate/TE solution (PLATE; prepared by combining 90 ml of sterile 45% PEG 3350, 10 ml of 1 M lithium acetate, 0.2 ml of 0.5 M EDTA and 1 ml Tris-HCl, pH 7.5) were combined in an microfuge tube along with a small swap of cells from a fresh plate or overnight culture. The mixture was quickly vortexed and left at room temperature for 24 hours at which time 100 µl was plated on selective media.

For spot dilution assays, cells were grown overnight in the indicated media, diluted to equal densities and then 3 µl of each culture and tenfold serial dilutions were spotted onto the indicated plates.

### Cdc34 expression and purification using bacteria

Cdc34ΔC^6XHis^ and Cdc34ΔC(S97A)^6XHis^ proteins, encoding the first 244 amino acids of Cdc34 fused to a 6XHis tag at their COOH-terminus, were expressed and purified for these studies. BL21(DE3) bacterial cells (Invitrogen, California USA) harboring plasmid AD002 (Cdc34ΔC^6XHis^) or pRC004 (Cdc34ΔC(S97A)^6XHis^) were grown in 15 ml of LB+ampicillin for 16 hours at 37°C. Five ml of the starter culture was used to inoculate 500 ml LB+ampicillin. The 500 ml culture was grown with vigorous shaking at 37°C for 2 hours or until the OD_600_ reached 1.2 at which time IPTG was added to a final concentration of 500 mM. The cells were allowed to grow for an additional 3 hours and were then collected by centrifugation. The bacterial pellet was resuspended in 5 ml of Lysis Buffer (50 mM NaH_2_PO_4_, 300 mM NaCl, 10 mM imidazole and one complete mini, EDTA-free protease inhibitor cocktail tablet (Roche, Mannheim, Germany) adjusted to pH 8 using NaOH). Five hundred µl of 10 mg/ml lysozyme was added and the cell suspension was sonicated for five cycles of ten seconds with a twenty-five percent duty cycle. The lysate was repeatedly drawn through a 23G1 needle to shear the DNA and then spun quickly in a clinical centrifuge. The supernatant was collected and mixed with 1.5 ml of Nickel Sepharose 6 Fast Flow resin (GE Healthcare-Biosciences, Pittsburgh, PA) that had been washed twice with Lysis Buffer. The nickel sepharose slurry was incubated on a rocker at 4°C for three hours. The beads were then washed twice with 1 ml of Wash Buffer (50 mM NaH_2_PO_4_, 300 mM NaCl, 20 mM imidazole adjusted to pH 8 with NaOH) for 1 hour at 4°C. Bound proteins were eluted by adding 500 µl of Elution Buffer (50 mM NaH_2_PO_4_, 300 mM NaCl, 250 mM imidazole adjusted to pH 8 with NaOH) and mixing at 4°C for 15 minutes. Two additional rounds of elution, accomplished by adding 750 µl of Elution Buffer to the beads and mixing at 4°C for 15 minutes, were necessary to recover the maximal amount of protein. The purified protein was then dialyzed overnight at 4°C in 1 L of Dialysis Buffer 1 (20 mM Tris-HCl pH 7.5, 2 mM EDTA, 4 mM MgCl_2_, 1 mM DTT, 55 mM NaCl and 20% glycerol) and for an additional hour in Dialysis Buffer 2 (20 mM Tris-HCl pH 7.5, 2 mM EDTA, 4 mM MgCl_2_, 1 mM DTT, 55 mM NaCl and 50% glycerol).

### ^Gst^Kinase over-expression and purification using yeast

An array of strain EJ758 [*MAT* **a** *his3-200, leu2-3,112, ura3-52, pep4::URA3*], harboring a unique plasmid expressing a single GST-kinase fusion gene under control of the P*_CUP1_* promoter [20] was used for determining Cdc34-S97 phosphorylation levels after single kinase over-expression (Table 2).

**Table 2:** Strains used in this study.

Strain	Genotype	Reference
5149	*MAT* **a** *vps34::Kan^R^ his3Δ1 leu2Δ0 met15Δ0 ura3Δ0*	[67]
3236	*MAT* **a** *vps15::Kan^R^ his3Δ1 leu2Δ0 met15Δ0 ura3Δ0*	[67]
3642	*MAT* **a** *gcn2::Kan^R^ his3Δ1 leu2Δ0 met15Δ0 ura3Δ0*	[67]
BL2	*MATα* prototroph	This study
EJ758(YIL035c)	*MAT* ***a*** * his3-Δ200 leu2-3,113 ura3-52 pep4::HIS3* pYEX4T-+rec::YIL035c	[20]
KS418	*MAT* **a**, *CDC34^tm^ ura3 leu2 trp1 lys2 ade2 ade3*	This study
KS422	*MAT* **a** *ura3 leu2 trp1 lys2 ade2 ade3*	[68]
KT945	*MATα his3 leu2 ura3 trp1 ade8 tpk2::HIS3 tpk3::TRP1*	[27]
RS13-58A-1	*MAT* **a** *his3 leu2 ura3 trp1 ade8 bcy1::LEU2 tpk1^w^ tpk2::HIS3 tpk3::TRP1*	[27]
KT1112	*MAT* **a** *leu2 ura3–52 his3*	[69]
KT1126	*MAT* **a** *leu2 ura3–52 bcy1–14*	[69]
RRC85	*MATα CDC34^tm^ (NAT1)*	[15]
YL10-1	*MAT* **a** *cdc34-2 leu2Δ1 ura3–52 trp1Δ63 his3Δ* Gal+	[13]
YL18	*MAT* ***a*** * cdc34::HIS3 ura3–52 leu2Δ1 trp1Δ63 his3Δ200 (pYL250)*	[13]

For analysis of Cdc34-S97 phosphorylation *in vivo*, each strain was grown overnight in 5 ml SD-Ura then diluted to 4×10^5^ cells/ml in 100 ml SD-Ura and grown for 8 hours at 30°C. After 8 hours of growth, copper sulfate was added to a final concentration of 500 µM to induce GST-Kinase expression. Induction lasted for 3 hours at which time the cells were pelleted in a Beckman Centrifuge at 4000×g, 4°C for 5 minutes and immediately frozen with liquid nitrogen. The level of Cdc34-S97 phosphorylation in each strain was measured qualitatively by western blot analysis.

To express and purify the ^GST^Kinase from yeast, a patch of cells was inoculated into 50 ml SD-Ura to an OD_600_ ∼0.2 and grown for the remainder of the day, shaking at 30°C. At the end of the day, the 50 ml culture was diluted into 500 ml so that it reached an OD_600_ of 0.8 the following morning. The following morning, copper sulfate was added to a final concentration of 500 µM to induce transcription of the ^GST^Kinase. The cultures were left to shake at 30°C for 2 hours. After 2 hr, the culture was split into 250 ml fractions and each was harvested by centrifugation in a Beckman Centrifuge at 4000×g. One tube was used for protein purification and the other was stored at −70°C for future purification. The cell pellet was resuspended in 1 ml EXTRACTION buffer (50 mM Tris-HCl (pH 7.5), 1 mM EDTA, 4 mM MgCl_2_, 5 mM DTT, 10% (v/v) glycerol, 1 M NaCl) with one protease inhibitor cocktail tablet (Roche Diagnostics, Germany) added per 10 ml buffer. Suspension was transferred to a microfuge tube and glass beads were added to the top of the tube. Cells were lysed at 4°C for 200 sec in a Biospec mini bead beater, using 10 cycles of shearing for 20 sec, followed by 1 minute cooling in an ice-water bath. The cell extract was removed from beads by puncturing the bottom of the tube with a flamed 25G needle and the extract was gently forced out with low-pressure air or light centrifugation. The remaining protein was washed off the beads with 0.5 ml EXTRACTION buffer. Two µl of 1.0 M PMSF was added to two ml of protein solution and insoluble cellular debris was removed by gentle centrifugation. The supernatant was transferred to a fresh microfuge tube and protein concentration was determined by the Bradford method [65].

The crude protein extract was diluted with an equal volume of No Salt Wash Buffer (50 mM Tris-HCl, 4 mM MgCl_2_, 1 mM DTT, 10% Glycerol) to bring the final salt concentration to 0.5 M NaCl. This salt concentration was chosen because it prevents most nonspecific protein-ligand or protein-protein interactions, while still preserving required functional interactions. 100 µl of pre-equilibrated glutathione-Sepharose resin were added per 2 ml of extract (as measured after addition of No Salt Wash Buffer). The tube was mixed gently at 4°C for 3 hours. To remove non-specific proteins, the mixture was centrifuged for 20–30 sec at low speed in a microfuge and the supernatant was decanted. The beads were washed twice with 1 ml of WASH BUFFER by mixing for 15 minutes, centrifuged and wash buffer was decanted. Bound proteins were eluted by adding 2 ml ELUTION BUFFER to the resin, followed by 1 hour of mixing, and then low speed centrifugation. The elution was dialyzed overnight against DIALYSIS BUFFER I (20 mM Tris-HCl, pH 7.4, 2 mM EDTA, 4 mM MgCl_2_, 1 mM dithiothreitol, 55 mM NaCl, 20% (v/v) glycerol) for 2 hours at 4°C and then overnight against DIALYSIS BUFFER II (20 mM Tris-HCl, pH 7.4, 2 mM EDTA, 4 mM MgCl_2_, 1 mM dithiothreitol, 55 mM NaCl, 50% (v/v) glycerol) and stored at −20°C. Normal ^GST^Kinase yield from a 250-ml culture is 0.5 ml of protein at ∼250 µg/ml.

### Antigen production and rabbit immunization

Antibodies were generated that recognize Cdc34 when serine 97 is phosphorylated (Open Biosystems, Alabama USA). The phosphopeptide DGRLCI(pS)ILHQ was synthesized and conjugated to Keyhole Limpet Hemocyanin (KLH). On day one, 500 µg of KLH-phosphopeptide conjugate was emulsified with Freund's complete adjuvant and subsequently used to immunize two New Zealand white rabbits. On days 14, 28 and 42 the rabbits were injected with an additional 250 µg of KLH-phosphopeptide conjugate emulsified with Freund's incomplete adjuvant. On days 35 and 56, approximately 25 ml of serum was collected from each rabbit. Production bleeds of 50 ml were taken from each rabbit on day 70 at which time the animals were euthanized.

### α-pS97 Antibody Purification

To ensure that the antibody specifically recognized Cdc34 phosphorylated on serine amino acid residue 97, 360 µg of purified, bacterially expressed Cdc34ΔC^6XHis^ was electrophoresed on a 10% SDS-PAGE gel. The protein was transferred to PVDF membrane for 2.5 hours at 30 volts at 4°C in Transfer Buffer (25 mM Tris, 190 mM Glycine, 15% methanol). The membrane was blocked with 5% milk in 1× KPBS-T for 1 hour at room temperature. After blocking, the membrane was exposed to 2 ml of crude anti-pS97 antisera which had been diluted with 13 ml of sterile 1× KPBS-T+0.02% sodium azide. The membrane was incubated overnight at 4°C with the antisera. The unbound fraction was collected and 11.5 of the 13 ml were purified with protein A beads as previously described [66]. α-pS97 antibodies were eluted from the protein A column with 2 ml of 100 mM Glycine (pH 2.2) and immediately neutralized with 100 µl of 1 M Tris-HCl (pH 9.1). An A_280_ measurement estimates the α-pS97 antibody concentration to be 1.7 mg/ml. Sodium azide was added to a final concentration of 0.02% prior to storage at 4°C.

### Yeast protein extraction methods

Once grown to the desired density, cells were collected by centrifugation for 5 minutes at 4000×g in a Beckman centrifuge at 4°C. The cell pellet was immediately frozen in liquid nitrogen and stored at −80°C until lysis. To extract the protein, the cell pellet was washed with water and then resuspended in 300 µl of Breaking Buffer (150 mM NaCl, 50 mM Tris-HCl pH7.5, 5 mM EDTA, 1% Triton X-100, 50 mM NaF and one Complete Mini protease inhibitor cocktail tablet (Roche Diagnostics, Germany) per 10 ml of breaking buffer). 500 µm acid-washed, glass beads (Sigma, USA) were added to the cell suspension and the cells were broken by repeated rounds of glass bead beating until a protein concentration of ∼5 µg/µl was achieved. Typically, this required three rounds of bead beating at one minute per round. Protein concentration was determined by the Bradford method [65].

The Horvath-Riezman protocol [19] is an alternate method for protein extraction from yeast cells. This protocol was used infrequently here but when used it has been denoted. Briefly, 3.75 µl of Extraction Buffer (60 mM Tris pH 6.8, 10% glycerol, 2% SDS, 5% β-mercaptoethanol, 5 mM EDTA, 50 mM NaF plus one Complete Mini protease inhibitor cocktail tablet (Roche Diagnostics, Germany) per 10 mL of extraction buffer) was added per milligram of wet cell pellet. Cells were resuspended in extraction buffer and boiled at 95°C for 5 min. Protein extract was spun at 14,000 rpm for 5 minutes at 4°C and the supernatant was saved as protein extract.

### SDS-PAGE and western blot analysis

5× Laemmli Buffer (20% glycerol, 2% SDS, 5% β-mercaptoethanol, 0.1% bromophenol blue and 62.5 mM Tris, pH 6.8) was added to the protein extract (∼50 µg of protein) made via the glass bead method. Protein from either extraction method was loaded in equal amounts onto a 10% or 12% SDS-PAGE gel. Separated proteins were transferred to a PVDF membrane using Transfer Buffer (25 mM Tris, 190 mM Glycine and 15% methanol). The membranes were washed with KPBS-T (135 mM NaCl, 2.5 mM KCl, 5.5 mM Na_2_HPO_4_, 1.5 mM KH_2_PO_4_, 0.2% Tween-20, pH to 7.2) and blocked with 5% milk in KPBS-T. Antibodies were used at the following dilutions, affinity purified α-Cdc34 (1∶10,000) and and α-TAP (1∶2000) (Open Biosystems, USA). Primary antibody was detected with an HRP conjugated goat α-rabbit secondary antibody at a 1∶10,000 dilution (Santa Cruz Biotechnology, USA).

### Detection of Cdc34 phosphorylation using ^32^P

Bacterially expressed Cdc34ΔC^6XHis^ and Cdc34(S97A)ΔC^6XHis^ (1.3 ηmoles) were incubated with 10 µl (1 U/µl) of bovine Protein Kinase A (Sigma Aldrich, USA) in PKA phosphorylation buffer (35 mM Potassium Phosphate, pH 7.4, 0.25 mg/ml BSA) plus 10 µl of 5× ATP solution (1 mM ATP, 25 mM MgCl_2_, 310 cpm/pmol [γ-^32^P]ATP) in a total reaction volume of 100 µl. When PKA Inhibitor (Sigma-Aldrich, USA) was used, 1 µl of 1 mg/ml solution was added to the reaction. The reactions were incubated at 30°C for 20 minutes and stopped by addition of 25 µl of 5× Laemmli Buffer. Samples were heated at 95°C for 3 minutes and 30 µl of each reaction was loaded onto a 12% SDS-PAGE gel. The gel was run and then dried and exposed to film for 4 hours. To quantitate the relative amounts of ^32^P incorporation, bands were cut from the dried gel and analyzed by a liquid scintillation counter.

### Detecting Cdc34 phosphorylation using α-pS97 antibody

Bacterially expressed Cdc34ΔC^6XHis^ (165 picomoles) was incubated with 10 µl (1 unit/µl) of bovine Protein Kinase A (Sigma Aldrich, USA) or 5 µl of ^GST^Tpk3 or ^GST^Sch9 purified from yeast in PKA phosphorylation buffer (35 mM Potassium Phosphate, pH 7.4, 0.25 mg/ml BSA) plus 10 µl of 5× ATP solution (1 mM ATP, 25 mM MgCl2) in a total reaction volume of 50 µl. When PKA Inhibitor (Sigma-Aldrich, USA) was used, 1 µl (1 mg/ml) was added to the reaction. The reactions were incubated at 30°C for 10 minutes and the samples were placed on ice. 10 µl of each was spotted onto a piece of PVDF membrane which had been pre-soaked in methanol. The spot was allowed to dry for 30 minutes. The membrane was re-wet with methanol and then processed as described in the western blotting section above.
